# SPATA33 localizes calcineurin to the mitochondria and regulates sperm motility in mice

**DOI:** 10.1073/pnas.2106673118

**Published:** 2021-08-26

**Authors:** Haruhiko Miyata, Seiya Oura, Akane Morohoshi, Keisuke Shimada, Daisuke Mashiko, Yuki Oyama, Yuki Kaneda, Takafumi Matsumura, Ferheen Abbasi, Masahito Ikawa

**Affiliations:** ^a^Research Institute for Microbial Diseases, Osaka University, Osaka 5650871, Japan;; ^b^Graduate School of Pharmaceutical Sciences, Osaka University, Osaka 5650871, Japan;; ^c^Graduate School of Medicine, Osaka University, Osaka 5650871, Japan;; ^d^The Institute of Medical Science, The University of Tokyo, Tokyo 1088639, Japan

**Keywords:** sperm motility, calcineurin, mitochondria, male fertility

## Abstract

Calcineurin is a calcium-dependent phosphatase that plays roles in a variety of biological processes including immune responses. In spermatozoa, there is a testis-enriched calcineurin composed of PPP3CC and PPP3R2 (sperm calcineurin) that is essential for sperm motility and male fertility. Because sperm calcineurin has been proposed as a target for reversible male contraceptives, identifying proteins that interact with sperm calcineurin widens the choice for developing specific inhibitors. Here, by screening the calcineurin-interacting PxIxIT consensus motif in silico and analyzing the function of candidate proteins through the generation of gene-modified mice, we discovered that SPATA33 interacts with sperm calcineurin via a PQIIIT sequence. *Spata33* knockout mice exhibit reduced sperm motility because of an inflexible midpiece, leading to impaired male fertility, which phenocopies *Ppp3cc* and *Ppp3r2* knockout mice. Further analysis reveals that sperm calcineurin disappears from the mitochondria in the *Spata33* knockout testis. In addition, immunoprecipitation analysis indicates that sperm calcineurin interacts with not only SPATA33 but also the mitochondrial protein VDAC2. These results indicate that SPATA33 localizes calcineurin to the mitochondria and regulates sperm motility.

## Significance

Calcineurin is a target of immunosuppressive drugs such as cyclosporine A and tacrolimus. In the immune system, calcineurin interacts with NFAT via the PxIxIT motif to activate T cells. In contrast, little is known about the proteins that interact with a testis-enriched calcineurin that is essential for sperm motility and male fertility. Here, we discovered that calcineurin interacts with SPATA33 via a PQIIIT sequence in the testis. Further analyses reveal that SPATA33 plays critical roles in sperm motility and male fertility. Our finding sheds new light on the molecular mechanisms of sperm motility regulation and the etiology of human male fertility. Furthermore, it may help us not only understand reproductive toxicities but also develop nonhormonal male contraceptives.

Fertilization is the union of two gametes, spermatozoa and eggs. In the female reproductive tract, spermatozoa need to travel a long distance to reach the egg and pass through the zona pellucida (ZP), an extracellular matrix that surrounds the egg. Sperm motility plays critical roles in these processes, which is executed by the flagellum that can be divided into three parts: midpiece, principal piece, and end piece (1, 2). The central motility apparatus of the flagellum is the axoneme, a “9+2” microtubule structure, that is found in all the parts of the flagellum. In addition to the axoneme, the midpiece possesses spirally arranged mitochondria called the mitochondrial sheath. The principal piece possesses a fibrous sheath that provides elastic rigidity and a scaffold for glycolytic and signaling molecules (1). The end piece contains no accessory structures. Defects in these structures’ formation or function could lead to impaired sperm motility and male infertility (1, 2).

Calcineurin is a calcium-dependent phosphatase that is evolutionarily conserved from yeasts to mammals and is comprised of two subunits, a catalytic and a regulatory subunit. In mammals, three isoforms (PPP3CA, PPP3CB, and PPP3CC) of the catalytic subunit and two isoforms (PPP3R1 and PPP3R2) of the regulatory subunit have been identified. *Ppp3ca*, *Ppp3cb*, and *Ppp3r1* are expressed ubiquitously and play roles in a variety of biological processes, including immune responses and cardiac morphogenesis (3, 4). In contrast, PPP3CC and PPP3R2 compose the testis-enriched calcineurin (sperm calcineurin), the disruption of which leads to an inflexible midpiece, impaired sperm motility, and male infertility (5–7). The administration of calcineurin inhibitors such as cyclosporine A and tacrolimus (FK506), both widely used immunosuppressant drugs, to wild-type (WT) males phenocopied the *Ppp3cc* and *Ppp3r2* knockout (KO) mice (5). These defects appear within 4 to 5 d of treatment, and male fertility recovered 1 wk after halting the drug administration, suggesting that sperm calcineurin can be a target for reversible and rapidly acting male contraceptives (5). However, it is challenging to develop molecules that specifically inhibit sperm calcineurin and not somatic calcineurin because of sequence similarities (82% amino acid identity between human PPP3CA and PPP3CC and 85% amino acid identity between human PPP3R1 and PPP3R2). Therefore, identifying proteins that interact with sperm calcineurin widens the choice of inhibitors that target the sperm calcineurin pathway.

The PxIxIT motif is a conserved sequence found in calcineurin-binding proteins (8, 9). Proteins with this consensus motif can be a substrate of calcineurin. For example, NFAT, in which the PxIxIT motif was first discovered, is translocated to the nucleus and activates gene expression when it is dephosphorylated by calcineurin, which plays critical roles in immune responses (10). In addition, PxIxIT motif-containing proteins can be regulators of calcineurin. For example, RCAN1 is an endogenous inhibitor of calcineurin (11, 12), while AKAP5 anchors calcineurin to the L-type Ca^2+^ channel in the plasma membrane (13, 14). In contrast to a variety of molecules known to bind to somatic calcineurin, little is known about the proteins that interact with sperm calcineurin.

Here, we searched the testis-enriched proteins that contain the PxIxIT consensus motif to identify substrates or regulators of sperm calcineurin, which may help not only reveal how sperm calcineurin works in regulating sperm motility but also discover new male contraceptive targets and understand reproductive toxicities that can be caused by calcineurin inhibitor–based immunosuppression. By analyzing the function of candidate proteins through the generation of gene-modified mice with the CRISPR/Cas9 system, we identified that SPATA33 is essential in localizing sperm calcineurin to the mitochondria and regulating sperm motility.

## Results

### Identification of Testis-Enriched Genes Encoding PxIxIT Motif.

**Fig. 1.**
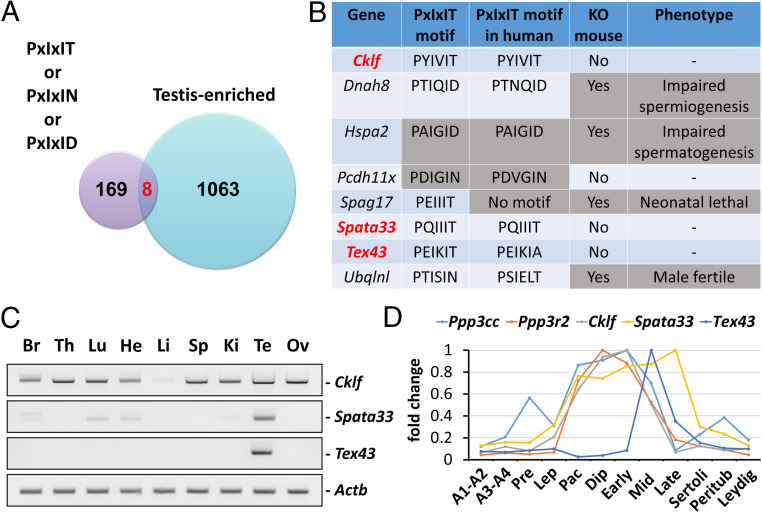
Screening proteins that interact with sperm calcineurin. (*A*) Mouse proteins with PxIxIT, PxIxIN, or PxIxID that are expressed strongly in the testis were searched. There are eight genes that are included in both categories. (*B*) Testis-enriched genes that possess PxIxIT motif. *Cklf*, *Spata33*, and *Tex43* were analyzed further. (*C*) RT-PCR of *Cklf*, *Spata33*, and *Tex43* using RNAs obtained from mouse various tissues. *Actb* was the control. Br: brain, Th: thymus, Lu: lung, He: heart, Li: liver, Sp: spleen, Ki: kidney, Te: testis, Ov: ovary. (*D*) In silico data analysis of *Ppp3cc*, *Ppp3r2*, *Cklf*, *Spata33*, and *Tex43* expression level. A1-A2: A1 and A2 differentiating spermatogonia, A3-A4: A3, A4, In, and B differentiating spermatogonia, Pre: preleptotene spermatocytes, Lep: leptotene/zygotene spermatocytes, Pac: pachytene spermatocytes, Dip: diplotene/secondary spermatocytes, Early: early round spermatids, Mid: mid round spermatids, Late: late round spermatids, Sertoli: Sertoli cells, Peritub: peritubular myoid cells, and Leydig: Leydig cells.

We searched mouse proteins that contain the PxIxIT (or PxIxIN or PxIxID) motif in silico and identified 169 proteins (Fig. 1*A*). We further searched genes expressed predominantly in the mouse testis (15) and found eight proteins, CKLF, DNAH8, HSPA2, PCDH11X, SPAG17, SPATA33, TEX43, and UBQLNL, that are included in both categories (Fig. 1 *A* and *B*). All the genes are conserved in humans. Among these genes, we omitted *Hspa2* and *Pcdh11x* because glycine is not preferred in the “x” of the PxIxIT motif (9). Furthermore, *Ubqlnl* was not analyzed because *Ubqlnl* KO male mice were fertile (15). KO mice of *Dnah8* and *Spag17* were also generated. *Dnah8* KO males were infertile because of impaired sperm flagellar formation (16). *Spag17* KO mice were neonatal lethal, likely due to impaired airway mucociliary clearance (17). A conditional KO approach using CRE recombinase reveals that *Spag17* is essential for the formation of sperm heads and flagella (18). It is still possible that DNAH8 or SPAG17 interacts with sperm calcineurin and regulates sperm motility, but we lowered the priority for these genes because KO mice were already generated, and their phenotypes were different from those of *Ppp3cc* and *Ppp3r2* KO mice. These results prompted us to analyze CKLF, SPATA33, and TEX43 further to discern if these proteins interact with sperm calcineurin.

RT-PCR was conducted to analyze the expression of *Cklf*, *Spata33*, and *Tex43* in mouse tissues (Fig. 1*C*). While *Cklf* was expressed ubiquitously, *Spata33* and *Tex43* were predominantly expressed in the testis. We then analyzed their expression in different testis cell types using a single-cell RNA sequencing database (Fig. 1*D*) (19). The expression of *Cklf* and *Spata33*, as well as sperm calcineurin (*Ppp3cc* and *Ppp3r2*), increased in pachytene spermatocytes. In contrast, *Tex43* expression increased in round spermatids. None of the genes were expressed strongly in testicular somatic cells such as Sertoli cells, peritubular myoid cells, or Leydig cells.

### *Cklf* Is Essential for Embryonic Development.

We generated *Cklf*, *Spata33*, and *Tex43* KO mice using the CRISPR/Cas9 system that has enabled us to analyze gene function in vivo in a short period of time (15, 20). For *Cklf*, we designed two guide RNAs (gRNAs) in exon 2 and two gRNAs in intron 4 to remove most of the coding region (*SI Appendix*, Fig. S1*A*). By introducing four gRNAs into the fertilized eggs with electroporation (21), we obtained mice that possessed a 6,257 deletion plus 1–base pair (bp) insertion (large deletion [LD] allele) (*SI Appendix*, Fig. S1 *A* and *B*). We mated heterozygous animals, but no homozygous mice were obtained (*SI Appendix*, Fig. S1*C*), indicating that *Cklf* is essential for embryonic development. Because this lethality hampered the analysis of the function of *Cklf* in mature spermatozoa, we lowered the priority to analyze *Cklf* further.

### *Tex43* Is Important for Normal Sperm Motility but Dispensable for Midpiece Flexibility.

For *Tex43*, we designed one gRNA in exon 1 (*SI Appendix*, Fig. S2*A*). By microinjecting the gRNA into fertilized eggs (22), we obtained a 4-bp deletion allele (*SI Appendix*, Fig. S2*B*), which resulted in a frameshift mutation of K13S with a premature stop codon introduced 33 amino acids later (*SI Appendix*, Fig. S2*C*). By conducting subsequent mating, we obtained *Tex43*^*-4/-4*^ mice that were viable and had no particular problems at first glance. We then performed mating tests and found that *Tex43*^−*4/*−*4*^ male mice sired a comparable number of pups to WT males (*SI Appendix*, Fig. S2*D*). To investigate if there are any slight abnormalities, we observed sperm morphology and analyzed sperm motility using the computer-assisted sperm analysis (CASA) system. Still, no abnormalities were observed in the sperm morphology of *Tex43*^−*4*/−*4*^ mice (*SI Appendix*, Fig. S3*A*), and the percentage of motile spermatozoa was comparable between *Tex43*^*WT*/−*4*^ and *Tex43*^−*4*/−*4*^ mice (*SI Appendix*, Fig. S3*B*). Velocity parameters such as average path velocity (VAP), straight-line velocity (VSL), and curvilinear velocity (VCL) were slightly decreased in the *Tex43* mutant spermatozoa (*SI Appendix*, Fig. S3*C*); however, the midpiece was not inflexible (*SI Appendix*, Fig. S3*D* and Movies S1 and S2), which is a characteristic of sperm calcineurin KO mice (5). These results indicate that TEX43 is involved in the regulation of sperm motility but is dispensable for male fertility.

### SPATA33 Is Essential for Midpiece Flexibility.

**Fig. 2.**
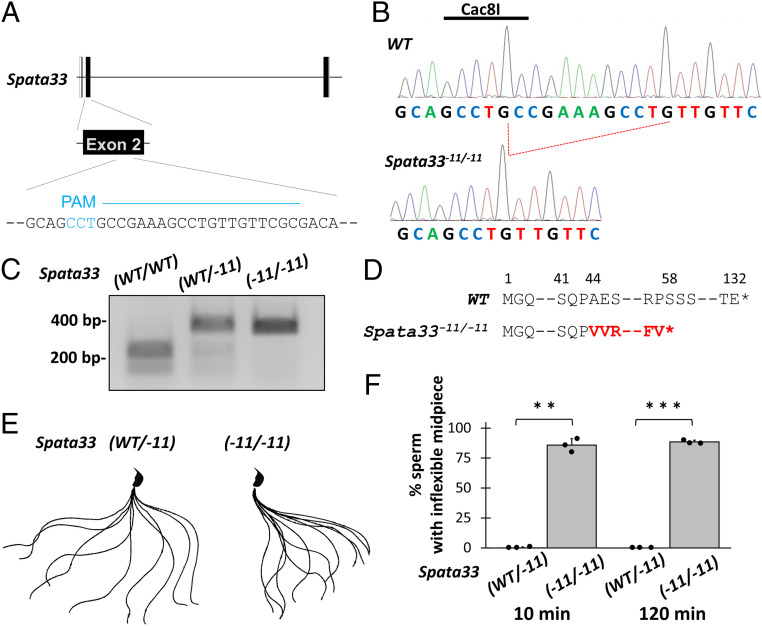
SPATA33 is essential for midpiece flexibility. (*A*) CRISPR/Cas9 targeting scheme. gRNA was designed within exon 2. Protospacer adjacent motif is shown in blue. (*B*) Wave pattern sequence of *Spata33*. In mutants, 11-bp nucleotides were deleted, which disrupts a Cac8I restriction site. (*C*) Genotyping *Spata33*^*−11*/−*11*^ mice by Cac8I digestion. (*D*) The 11-bp deletion caused a A44V mutation with a premature stop codon introduced 14 amino acids later. (*E*) Flagellar waveforms were analyzed 10 min after incubation. Single frames throughout one beating cycle were superimposed. Spermatozoa from *Spata33*^*−11*/−*11*^ mice exhibit inflexible midpieces. (*F*) The percentage of the spermatozoa with an inflexible midpiece at 10 and 120 min after sperm suspension. *n* = 3 males each for *Spata33*^*WT*/−*11*^ and *Spata33*^*−11*/−*11*^ mice. **P < 0.01 and ***P < 0.001 (Student's *t* test).

For *Spata33*, we designed one gRNA in Exon 2 (Fig. 2*A*) and microinjected it into the fertilized eggs (B6D2F1 × B6D2F1). We obtained an 11-bp deletion allele (Fig. 2*B*) confirmed with genomic PCR and subsequent digestion by the restriction enzyme, Cac8I. (Fig. 2*C*). The 11-bp deletion resulted in a frameshift mutation of A44V with a premature stop codon introduced 14 amino acids later (Fig. 2*D*). By subsequent mating, S*pata33*^−*11*/−*11*^ mice were obtained that exhibited no overt abnormalities.

**Fig. 3.**
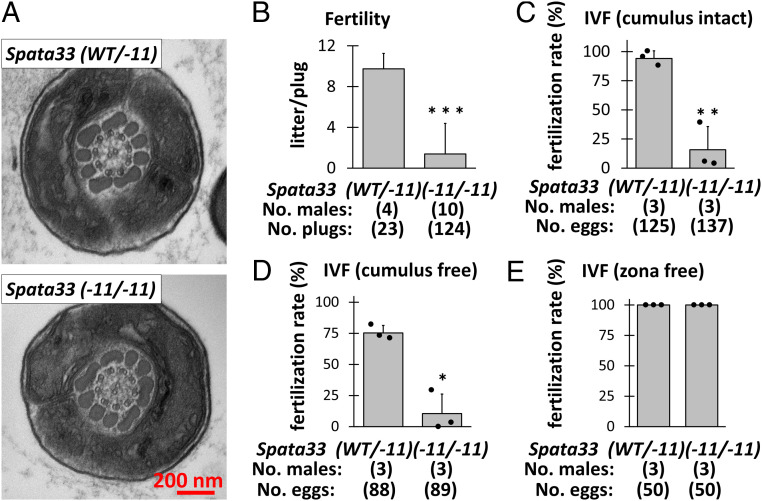
In vivo and in vitro fertility of *Spata33*^*−11*/−*11*^ mice. (*A*) No obvious ultrastructural abnormalities were observed in the midpiece of *Spata33*^*−11*/−*11*^ spermatozoa. (*B*) Number of litters born per plug detected. (*C*) In vitro fertilization (IVF) with cumulus-intact oocytes. (*D*) IVF with cumulus-free oocytes. (*E*) IVF with zona-free oocytes. The impaired fertility of *Spata33*^*−11*/−*11*^ mice was rescued. *P < 0.05, **P  <  0.01, and ***P  <  0.001 (Student's *t* test).

First, we analyzed sperm motility using the CASA system. Although there was no difference in the percentage of motile spermatozoa, velocity parameters such as VAP, VSL, and VCL decreased in the spermatozoa from *Spata33*^−*11*/*−11*^ mice (*SI Appendix*, Fig. S4). We then analyzed waveform patterns of the flagella. We found that the midpiece was inflexible in *Spata33*^−*11*/−*11*^ mice (Fig. 2 *E* and *F* and Movies S3 and S4), which was similarly observed in sperm calcineurin KO spermatozoa (5). We further carefully observed sperm morphology; however, no structural abnormalities were observed in the testis (*SI Appendix*, Fig. S5*A*), spermatozoa (*SI Appendix*, Fig. S5*B*), or ultrastructure of the midpiece (Fig. 3*A* and *SI Appendix*, Fig. S5*C*), which is consistent with sperm calcineurin KO spermatozoa (5).

To further analyze the phenotype of *Spata33*^−*11*/−*11*^ mice, we performed mating tests and found that the fertility of *Spata33*^−*11*/−*11*^ male mice was severely impaired (Fig. 3*B* and *SI Appendix*, Fig. S5*D*). We also performed in vitro fertilization and discovered that fertilization rates were significantly reduced in *Spata33*^−*11*/−*11*^ mice (Fig. 3*C*). Removing cumulus cells did not rescue the impaired fertilization rates (Fig. 3*D*). In contrast, removing both cumulus cells and the ZP rescued fertilization rates (Fig. 3*E*), indicating that penetration through the ZP was impaired in the *Spata33* mutant spermatozoa, likely due to abnormal sperm motility consistent with sperm calcineurin KO spermatozoa (5).

### Sperm Calcineurin Recognizes PQIIIT in SPATA33.

**Fig. 4.**
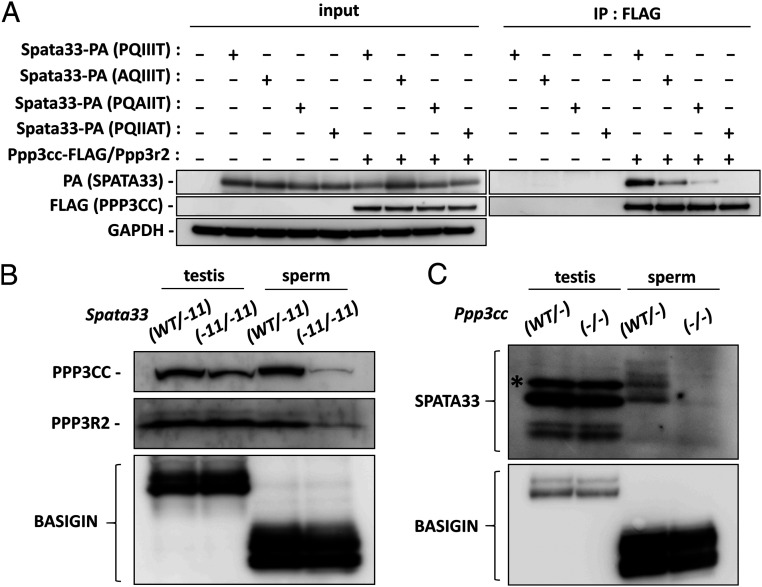
SPATA33 interacts with sperm calcineurin via PQIIIT sequence. (*A*) *Spata33-PA* (PQIIIT) or mutated *Spata33-PA* (AQIIIT, PQAIIT, or PQIIAT) was coexpressed with *Ppp3cc-FLAG*/*Ppp3r2* in HEK293T cells, and immunoprecipitation (IP) with FLAG M2 antibody was performed. GAPDH was the control. (*B*) PPP3CC and PPP3R2 decreased in the spermatozoa from *Spata33*^*−11*/−*11*^ mice. BASIGIN was the control. (*C*) SPATA33 decreased in the spermatozoa from *Ppp3cc*^−/−^ mice. BASIGIN was the control. The asterisk indicates a nonspecific band.

Similar phenotypes in *Spata33*^−*11*/−*11*^ mice and sperm calcineurin KO mice suggest an interaction between SPATA33 and sperm calcineurin. We coexpressed sperm calcineurin and SPATA33 in human embryonic kidney 293T (HEK293T) cells and performed an immunoprecipitation analysis (Fig. 4*A* and *SI Appendix*, Fig. S6*A*). FLAG-tagged PPP3CC was pulled down using a FLAG antibody, and after Western blotting analysis, we found that PA-tagged SPATA33 precipitated down as well (Fig. 4*A*). In contrast, when we substituted a PQIIIT sequence (the PxIxIT motif) of SPATA33 to AQIIIT, PQAIIT, or PQIIAT, the amount of precipitated PA-tagged SPATA33 was decreased or diminished, indicating that SPATA33 interacts with PPP3CC via the PQIIIT sequence. We confirmed that sperm calcineurin interacts with WT SPATA33 containing a PQIIIT sequence but not with mutated SPATA33 containing a PQIIAT sequence by reciprocal immunoprecipitation analysis (*SI Appendix*, Fig. S6*A*). This PQIIIT sequence is conserved in mammals (*SI Appendix*, Fig. S6*B*), suggesting that sperm calcineurin could bind to SPATA33 in other species as well. By coexpressing human calcineurin and SPATA33 in HEK293T cells, we confirmed that human SPATA33 can interact with not only human sperm calcineurin but also somatic calcineurin (*SI Appendix*, Fig. S6*C*).

To further analyze the relationship between SPATA33 and sperm calcineurin, we examined the amount of PPP3CC and PPP3R2 in *Spata33*^−*11*/−*11*^ mice. We found that the amounts of both proteins were significantly lower in the *Spata33* mutant spermatozoa than that of the control spermatozoa but not in the testis (Fig. 4*B* and *SI Appendix*, Fig. S6*D*). We then generated an antibody against SPATA33 to analyze the protein in *Ppp3cc* KO testis and spermatozoa. The generated antibody detected one major band around 19 kDa and some minor bands in both control testis and spermatozoa, which were not found in the *Spata3*3^−*11*/−*11*^ mice (*SI Appendix*, Fig. S6*E*). In contrast, the 20 kDa signal in testis remained even in *Spata3*3^−*11*/−*11*^ mice, which may be a nonspecific band. Using this antibody, we found that SPATA33 disappeared in the *Ppp3cc* KO spermatozoa, while SPATA33 was detected in the *Ppp3cc* KO testis (Fig. 4*C* and *SI Appendix*, Fig. S6*F*). These results indicate that SPATA33 and sperm calcineurin need to interact with each other to maintain the proper amount of these proteins in the mature spermatozoa.

### Inflexible Midpiece Was Not Due to Off-Target Effects.

There are some cases in which alternative splicing variants remain functional after an indel mutation. The off-target cleavages also compromise gene function analysis. To overcome this issue, we generated mutant mice that had excised a significant portion of the gene (large deletion “LD”) using different gRNAs. Two gRNAs targeting exon 1 and exon 3 were electroporated into fertilized C57BL/6N mouse eggs, and LD mice with a deletion of 9,087 bp were obtained (*SI Appendix*, Fig. S7 *A* and *B*). Through Western blotting analysis, we confirmed that the LD mice had the same band pattern as the 11-bp deletion mice (*SI Appendix*, Fig. S7*C*), which reconfirmed that SPATA33 is absent in both lines, and the 20 kDa signal in the testis is nonspecific. The fertility of *Spata33*^*LD*/*LD*^ male mice was severely impaired (*SI Appendix*, Fig. S7*D*), and their spermatozoa exhibited inflexible midpiece (*SI Appendix*, Fig. S7*E* and Movies S5 and S6), even though sperm morphology was normal (*SI Appendix*, Fig. S7*F*). Sperm motility analysis via the CASA system showed that all the velocity parameters were impaired in the *Spata33*^*LD*/*LD*^ mice compared to the control mice, consistent with *Spata33*^*−11*/−*11*^ mice (*SI Appendix*, Fig. S8). These results confirm that the disruption of SPATA33 resulted in midpiece inflexibility.

### SPATA33 Is Essential to Localize Sperm Calcineurin to the Midpiece.

**Fig. 5.**
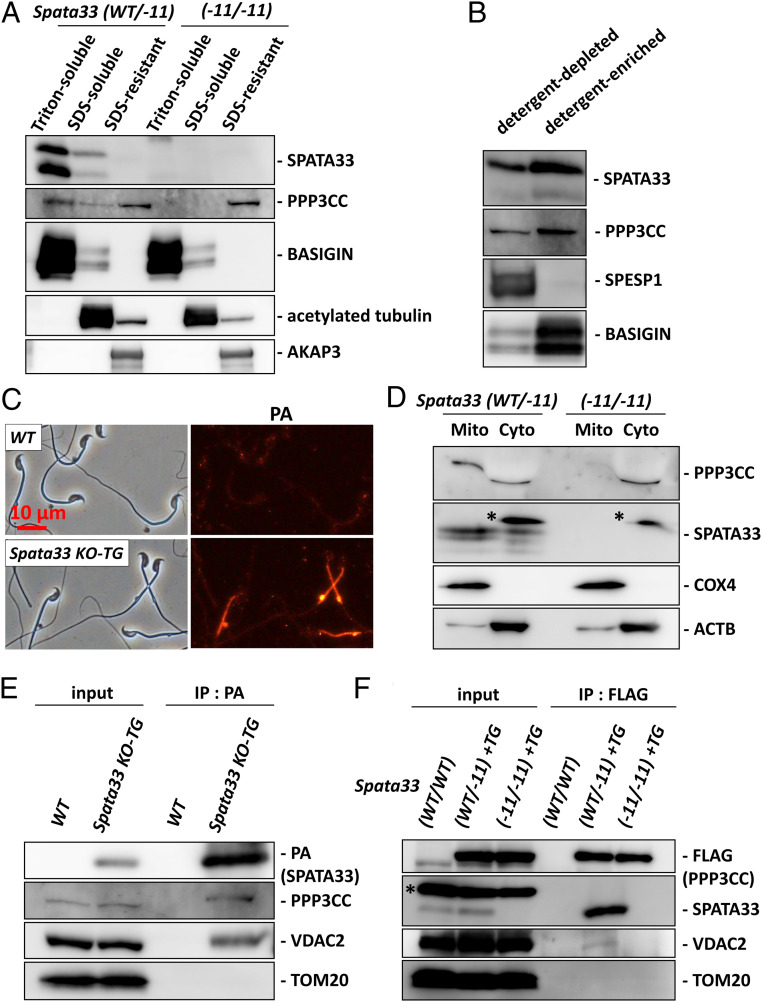
SPATA33 localizes sperm calcineurin to the mitochondria. (*A*) Fractionation of mouse spermatozoa. SPATA33 was found in the Triton-soluble fraction. PPP3CC is found in both the Triton-soluble and SDS-resistant fraction. Only PPP3CC in the Triton-soluble fraction was depleted in *Spata33*^*−11*/*−11*^ mice. BASIGIN, acetylated tubulin, and AKAP3 were used as makers for the Triton-soluble, SDS-soluble, and SDS-resistant fractions, respectively. (*B*) Phase separation of Triton X-114 extracts of spermatozoa. SPATA33 and PPP3CC were enriched in the detergent-enriched phase. SPESP1 is a marker for the detergent-depleted fraction, whereas BASIGIN is a marker for the detergent-enriched fraction. (*C*) SPATA33-PA localization was analyzed using *Spata33-PA* TG mice. SPATA33-PA is localized in the midpiece. (*D*) Separation of mitochondrial and cytosolic fractions of testicular proteins. Both PPP3CC and SPATA33 were found in the mitochondrial fraction. PPP3CC was depleted in the mitochondrial fraction of *Spata33*^*−11*/*−11*^ mice. PPP3CC bands in the cytosolic fraction were shifted due to the existence of a large amount of protein (probably tubulin). The asterisks indicate nonspecific bands. (*E*) Immunoprecipitation with anti-PA antibody was performed using testis lysates of SPATA33-PA TG mice. PPP3CC and VDAC2 were immunoprecipitated with SPATA33-PA. (*F*) Immunoprecipitation with anti-FLAG antibody was performed using testis lysates of *Ppp3cc-FLAG* TG mice. SPATA33 and VDAC2 were immunoprecipitated with PPP3CC-FLAG. The asterisk indicates a nonspecific band.

To understand why the amount of sperm calcineurin is lower in the spermatozoa of *Spata33*^−*11*/−*11*^ mice when the whole-sperm proteins were extracted with a strong urea lysis buffer (Fig. 4*B* and *SI Appendix*, Fig. S6*D*), we analyzed their localization in spermatozoa in more detail using different lysis buffers. We separated sperm proteins into three fractions, a Triton X-100–soluble fraction containing membrane-associated and cytosolic proteins, a Triton X-100–resistant/SDS-soluble fraction containing axonemal proteins, and a Triton X-100–resistant/SDS-resistant fraction containing proteins associated with a fibrous sheath and an outer dense fiber (Fig. 5*A* and *SI Appendix*, Fig. S9*A*) (23, 24). After performing Western blotting analysis, we found that SPATA33 is mainly extracted in the Triton X-100–soluble fraction. In contrast, PPP3CC is extracted in both the Triton X-100–soluble fraction and Triton X-100–resistant/SDS-resistant fraction. When we analyzed *Spata33*^−*11*/−*11*^ mouse spermatozoa, PPP3CC disappeared in the Triton X-100–soluble fraction but not in the Triton X-100–resistant/SDS-resistant fraction (Fig. 5*A* and *SI Appendix*, Fig. S9*A*). These results indicate that there are two populations of sperm calcineurin in the mature spermatozoa, a Triton X-100–soluble fraction and a Triton X-100–resistant/SDS-resistant fraction, and SPATA33 is vital to localize sperm calcineurin to the Triton X-100–soluble fraction, a membrane-associated, or cytosolic fraction. We then separated sperm proteins with Triton X-114, which enriches membrane-associated proteins (25). Both SPATA33 and PPP3CC were detected in the Triton X-114–enriched fraction (Fig. 5*B*), suggesting that both proteins are associated with membranes.

To analyze where in the membrane fraction SPATA33 and sperm calcineurin are localized, we generated transgenic (TG) mice that express PA-tagged SPATA33 under the testis-specific *Clgn* promoter (*SI Appendix*, Fig. S9*B*) (26) because we could not obtain a SPATA33 antibody that works for immunofluorescence. We detected SPATA33-PA in both the testis and spermatozoa of TG mice by Western blotting (*SI Appendix*, Fig. S9*C*). Furthermore, the transgene rescued both the decreased amount of PPP3CC (*SI Appendix*, Fig. S9*C*) and impaired male fertility (*SI Appendix*, Fig. S9*D*) in *Spata33*^−*11*/−*11*^ mice, indicating that the transgene is functional. By performing immunofluorescence analysis with a PA antibody, we revealed that SPATA33-PA was localized to the midpiece (Fig. 5*C*), indicating that SPATA33 is vital to localize sperm calcineurin in the midpiece. In previous studies, it was shown that PPP3CC and PPP3R2 were localized in both midpiece and principal piece with a stronger signal in the midpiece (5, 6). We confirmed that strong PPP3R2 signal in the midpiece disappeared in the mature spermatozoa of *Spata33*^−*11*/−*11*^ mice (*SI Appendix*, Fig. S9*E*).

### SPATA33 Localizes Sperm Calcineurin to the Mitochondria via VDAC2.

There are two membrane fractions in the sperm midpiece, the mitochondrial membrane and the plasma membrane. Because mitochondria are located in the midpiece, consistent with SPATA33 localization (Fig. 5*C*), we isolated mitochondrial proteins from the testis. Both PPP3CC and SPATA33 were found in the mitochondrial fraction. In addition, PPP3CC in the mitochondrial fraction was depleted in the *Spata33*^−*11*/−*11*^ testis (Fig. 5*D* and *SI Appendix*, Fig. S9*F*), suggesting that SPATA33 localizes sperm calcineurin to the mitochondria. Very recently, it was reported that SPATA33 is localized in the mitochondria by interacting with the mitochondrial outer membrane protein, VDAC2 (27). We then performed an immunoprecipitation analysis using TG mice that express PA-tagged SPATA33 in the testis (*SI Appendix*, Fig. S9*C*) and confirmed that SPATA33-PA interacts with both PPP3CC and VDAC2 but not with another mitochondrial outer membrane protein, TOM20 (Fig. 5*E*). Furthermore, we performed an immunoprecipitation analysis using TG mice that express FLAG-tagged PPP3CC in germ cells (5). When we pulled down PPP3CC with an anti-FLAG antibody, SPATA33 and VDAC2 were detected (Fig. 5*F*). In addition, the interaction between PPP3CC and VDAC2 was not detected when *Spata33*^−*11*/−*11*^ testis was used (Fig. 5*F*). These results indicate that SPATA33 mediates the interaction of sperm calcineurin and VDAC2.

## Discussion

In this study, we screened testis-enriched proteins that contain a calcineurin-interacting consensus motif (PxIxIT, PxIxIN, or PxIxID) and identified that SPATA33 interacts with sperm calcineurin via a PQIIIT sequence. Further analyses revealed that SPATA33 is important in localizing sperm calcineurin to the mitochondria and regulating sperm motility. It has been shown that sperm calcineurin is localized in both the midpiece and principal piece (5, 6). Because *Spata33* mutant spermatozoa exhibit inflexible midpieces, our results suggest that sperm calcineurin in the mitochondria regulates midpiece flexibility. In contrast, a Triton X-100–resistant/SDS-resistant fraction of sperm calcineurin, likely in the principal piece (such as the fibrous sheath) or both midpiece and principal piece (such as the axoneme or outer dense fiber), remains in the *Spata33* mutant spermatozoa. It has been shown that calcineurin is firmly bound to the dynein of the axoneme and is also localized in the fourfold Ca^2+^ signaling domain (28–30). Sperm calcineurin in these localizations may be associated with the Triton X-100–resistant/SDS-resistant fraction and involved in regulating sperm motility and/or function.

Because the administration of calcineurin inhibitors such as cyclosporine A and FK505 phenocopied the *Ppp3cc* and *Ppp3r2* KO mice (5), phosphatase activity of sperm calcineurin is important to regulate midpiece flexibility, and there must be substrates that are dephosphorylated by sperm calcineurin. Because spermatozoa are thought to be transcriptionally inactive during the epididymal transit when sperm calcineurin works (5), it is unlikely that NFAT, a transcriptional factor that is a well-known calcineurin substrate, is a target of sperm calcineurin. Among the genes that are listed in this study (Fig. 1*B*), we could not analyze the function of CKLF in the testis because KO mice were embryonic lethal. However, because a part of the PxIxIT motif of CKLF is predicted to be included in the transmembrane region, it probably is difficult for sperm calcineurin to bind to CKLF. We also cannot exclude the possibility that DNAH8 or SPAG17 is essential for not only flagellar formation (16, 18) but also midpiece flexibility; however, because DNAH8 and SPAG17 are axonemal proteins, it is unlikely that sperm calcineurin in the mitochondria directly binds to and dephosphorylates these proteins to regulate midpiece flexibility.

Because we reveal that sperm calcineurin is localized in the mitochondria via SPATA33, it is likely that substrates are associated with this organelle to regulate midpiece flexibility. One candidate substrate may be VDAC2 because it interacts with sperm calcineurin. Interestingly, it has been reported that there are phosphorylation sites in VDAC2 (31). In addition, a recent study suggests that VDAC2 is used as a scaffold for molecules that regulate sperm mitochondrial dynamics and adherence (32). Sperm calcineurin may modulate these molecules and regulate the interaction between mitochondria, which results in the midpiece flexibility. In addition to the mitochondrial interaction, it is also possible that sperm calcineurin regulates the interaction between the mitochondria and outer dense fiber. Intriguingly, it has been reported that breaking down the attachment between the mitochondria and outer dense fibers is necessary for the midpiece to be motile (33). A different approach outside of the screening PxIxIT motif, such as mass spectrometry analysis after immunoprecipitation or biotin proximity labeling (34), can be an excellent way to identify the substrates.

While *Ppp3cc* and *Ppp3r2* KO males were completely infertile (5), *Spata33*^−*11*/−*11*^ male mice had partial fertility. This difference may be caused by a Triton X-100–resistant/SDS-resistant fraction of sperm calcineurin that remains in the *Spata33* mutant spermatozoa. Although it remains to be determined if inhibiting the interaction of sperm calcineurin and SPATA33 could cause infertility in humans, it could be one option of a combinational contraceptive strategy that targets multiple steps of fertilization. Furthermore, when developing immunosuppressive drugs that target the calcineurin–NFAT interaction via the PxIxIT motif (10, 35), our results indicate that potential adverse effects on male fertility may result because of the inhibition of the calcineurin–SPATA33 interaction via the same motif.

In summary, we identified the calcineurin-binding protein with the PxIxIT motif that localizes calcineurin to the mitochondria. Revealing how SPATA33 and sperm calcineurin regulate midpiece flexibility could shed light on understanding male infertility and may lead to the development of nonhormonal male contraceptives. Furthermore, this unique function of SPATA33 localizing calcineurin to the mitochondria may be utilized by somatic calcineurin, considering that there is a weak expression of *Spata33* in the brain, lung, and heart.

## Materials and Methods

### Animals.

All animal experiments were approved by the Animal Care and Use Committee of the Research Institute for Microbial Diseases, Osaka University. Mice were purchased from CLEA Japan or Japan SLC. All gene-modified mice generated in this study will be made available through either the RIKEN BioResource Research Center or the Center for Animal Resources and Development (CARD), Kumamoto University.

### Identification of Testis-Enriched Protein with PxIxIT Motif.

For identifying proteins with the PxIxIT motif, mouse proteome data were obtained from Uniprot (https://www.uniprot.org/). Using grep command (UNIX), texts including P*I*IT, P*I*IN, or P*I*ID were searched. Testis-enriched proteins were identified using the UniGene EST database as described previously (15). A total of 39 proteins containing the PxIxID motif, 45 proteins containing PxIxIN, 84 proteins containing PxIxIT, and 1 protein containing both PxIxID and PxIxIN were found.

### RT-PCR.

RNA was prepared from multiple adult tissues of C57BL/6N mice using TRIzol (Thermo Fisher Scientific) according to the manufacturer's protocol. The obtained RNA was reverse transcribed to complementary DNA (cDNA) with the SuperScript III first-strand synthesis system (Thermo Fisher Scientific) using an oligo (dT) primer. PCR was then performed using 10 ng cDNA with the primers listed in *SI Appendix*, Table S1. The amplification conditions for the subsequent PCR were 1 min at 94 °C, followed by 35 cycles of 94 °C for 30 s, 65 °C for 30 s, and 72 °C for 30 s, with a final 2 min extension at 72 °C.

### In Silico Expression Analysis.

Published single-cell transcriptome data of the mouse testis (19) was obtained. The expression of each gene in these cells was analyzed using the Loupe Cell Browser 3.3.1 (10× Genomics).

### Generation of *Tex43* and *Spata33* Mutant Mice (indel).

Mutant mice (indel) were generated as described previously (22). B6D2F1 females were superovulated and mated with B6D2F1 males, and fertilized eggs were collected from the oviduct. Circular pX330 plasmids were injected into one of the pronuclei at 5 ng/µL. The sequences of gRNA used were listed in *SI Appendix*, Table S1. The injected zygotes were cultured in potassium simplex optimization medium (KSOM) (36), and two-cell embryos were transferred into the oviduct of pseudopregnant Institute of Cancer Research (ICR) mice the next day. Obtained pups were genotyped by performing PCR and subsequent Sanger sequencing. Primers used for genotyping were listed in *SI Appendix*, Table S1.

### Generation of *Cklf* and *Spata33* Mutant Mice (LD).

Mutant mice (LD) were generated as described previously (21). Fertilized eggs were obtained by B6D2F1 × B6D2F1 (*Cklf*) or C57BL/6N × C57BL/6N (*Spata33*) mating. The CRISPR/Cas9 complex was generated by incubating CRISPR RNAs (crRNAs) (Sigma-Aldrich), trans-activating crRNA (tracrRNA) (#TRACRRNA05N-5NMOL, Sigma-Aldrich), and CAS9 protein (#A36497, Thermo Fisher Scientific) at 37 °C for 5 min. The complex was then electroporated into fertilized eggs using a super electroporator NEPA21 (NEPA GENE) (poring pulse, voltage: 225 V, pulse width: 2 ms, pulse interval: 50 ms, and number of pulses: +4; transfer pulse, voltage: 20 V, pulse width: 50 ms, pulse interval: 50 ms, and number of pulses: ±5). The zygotes were cultivated in KSOM medium, and two-cell embryos were transferred into the oviduct of pseudopregnant ICR mice the next day. Obtained pups were genotyped by performing PCR and subsequent Sanger sequencing. Primers used for genotyping were listed in *SI Appendix*, Table S1.

### Generation of *Spata33* TG Mice.

Mouse *Spata33* cDNA-PA tag with a rabbit polyA signal under the mouse *Clgn* promoter (*SI Appendix*, Fig. S9*B*) was prepared. The linearized DNA was injected into one of the pronuclei of fertilized eggs that were obtained by B6D2F1 × B6D2F1 mating, and the zygotes were cultivated and transferred into females as mentioned above.

### Mating Tests.

Sexually matured male mice were individually caged with two or three 8-wk-old B6D2F1 female mice for 2 mo, and plugs were checked every morning. The number of pups was counted on the day of birth.

### Sperm Motility Analysis.

Sperm motility was analyzed as described previously (5, 37). Spermatozoa obtained from the cauda epididymis were incubated in a 100 µl drop of Toyoda, Yokoyama, and Hoshi (TYH) medium (38). After an incubation period of 10 and 120 min in TYH medium, spermatozoa were obtained from the top of the drops and analyzed using the CEROS (Version 12.3; Hamilton Thorne Biosciences) (*SI Appendix*, Figs. S3 *B* and *C* and S4) or CEROS II (software version 1.4; Hamilton Thorne Biosciences) (*SI Appendix*, Fig. S8) sperm analysis system. For waveform tracing, spermatozoa were observed with an Olympus BX-53 microscope equipped with a high-speed camera (HAS-L1, Ditect). The motility was videotaped at 200 frames per second and analyzed for waveforms using the sperm motion analyzing software (BohBohsoft) (39).

### Transmission Electron Microscopy.

The cauda epididymis samples were prepared for transmission electron microscopy as described previously (40). Sections were observed using a JEM-1400 plus electron microscope (JEOL) at 80 kV with a CCD Veleta 2K × 2K camera (Olympus).

### In Vitro Fertilization.

In vitro fertilization was performed as described previously (41). Spermatozoa were collected from the cauda epididymis and incubated in a TYH drop for 120 min at 37 °C under 5% CO_2_. Eggs that were collected from superovulated females were treated with 330 µg/mL hyaluronidase (Sigma-Aldrich) for 10 min to remove the cumulus cells (cumulus-free eggs) or with 1 mg/mL collagenase (Sigma-Aldrich) for 10 min to remove the ZP (zona-free eggs). The spermatozoa were then added to the TYH drop containing intact, cumulus-free, or zona-free eggs at a final density of 2 × 10^5^ spermatozoa/mL. For intact or cumulus-free eggs, two-cell embryos were counted the next day. For zona-free eggs, the formation of pronuclei was observed 6 h after insemination.

### Periodic Acid–Schiff Staining of Testis Sections.

A histological analysis of testis sections was performed as previously described (42). The sections were observed with an Olympus BX-53 microscope.

### Generation of SPATA33 Recombinant Proteins.

Mouse *Spata33* or human *SPATA33* was amplified from mouse or human testis cDNA and ligated into the PA-tagged (C terminus) or 1D4-tagged (C terminus) pCAG vector that contains the CAG promoter and a rabbit globin poly (A) signal (43). Primers used are listed in *SI Appendix*, Table S1. Mutated moues *Spata33* was prepared utilizing the Sma1 restriction site located near the PQIIIT sequence. The *Spata33* expression vectors were cotransfected with the expression vectors of calcineurin (5) transiently into HEK293T cells and the cells were cultured for 24 h before collection. The cells were then lysed with a solution containing 1% Triton X-100, 50 mM NaCl, 20 mM Tris HCl, pH 7.4, and protease inhibitor mixture (#25955, Nacalai Tesque), incubated for 30 min at 4 °C, and centrifuged at 15,300 *g* for 15 min at 4 °C to collect supernatants.

### Immunoprecipitation.

The lysates were incubated for 60 min at 4 °C with anti-FLAG antibody (#F1804, Sigma-Aldrich) or anti-PA antibody (#012–25863, FUJIFILM Wako Pure Chemical) conjugated Dynabeads (#10009D, Thermo Fisher Scientific). The immune complexes were washed three times with a solution containing 40 mM Tris HCl, 150 mM NaCl, 0.1% Triton X-100, and 10% glycerol and eluted with sample buffer (66 mM Tris HCl, 2% SDS, 10% glycerol, and 0.005% Bromophenol Blue).

### Sperm Protein Fractionation with Different Lysis Buffers.

Sperm protein fractionation was performed as described previously (23, 24). Spermatozoa were obtained from the cauda epididymis, suspended in 1% Triton X-100 lysis buffer (50 mM NaCl, 20 mM Tris HCl, pH 7.5, and protease inhibitor mixture), and incubated for 2 h at 4 °C. The sample was centrifuged at 15,000 *g* for 10 min to separate the Triton-soluble fraction (supernatant) and the Triton-resistant fraction (pellet). The pellet was then resuspended in 1% SDS lysis buffer (75 mM NaCl and 24 mM ethylenediaminetetraacetic acid, pH 6.0), incubated for 1 h at room temperature, and centrifuged at 15,000 *g* for 10 min to separate the SDS-soluble fraction (supernatant) and SDS-resistant fraction (pellet). The pellet was resuspended in sample buffer, boiled for 5 min, and centrifuged at 15,000 *g* for 10 min.

### Phase Separation of Sperm Triton X-114 Extracts.

Spermatozoa were collected from the cauda epididymis into phosphate-buffered saline (PBS) and centrifuged at 1,000 *g* for 5 min at 4 °C. The sperm pellets were suspended in PBS containing 1% Triton X-114 and protease inhibitor mixture (#25955, Nacalai Tesque). The sperm suspensions were placed on ice for 1 h with occasional vortexing. After centrifuging at 15,300 *g* for 30 min at 4 °C, the supernatants were collected. After incubation at 37 °C for 15 min, the tubes were centrifuged at 600 *g* for 15 min at room temperature to separate the detergent-depleted phase and detergent-enriched phase. PBS was added to resuspend the detergent-enriched phase. Both detergent-depleted and detergent-enriched phases were mixed with sample buffer.

### Mitochondria Isolation from Testes.

Mitochondria were isolated from testes using a mitochondria isolation kit for tissue (#89801, Thermo Fisher Scientific) following the manufacturer’s protocol (32).

### Antibody Generation.

Rabbit polyclonal antibody was produced by immunization with mouse SPATA33 polypeptide (C plus DKESQPAESLLFATSKHSR). Generated SPATA33 antibody was purified using the SPATA33 polypeptide and SulfoLink coupling resin (Thermo Fisher Scientific).

### Western Blotting.

Western blotting analysis was performed as described previously (21). For extracting whole proteins from testis or spermatozoa (no fractionation), urea lysis buffer (6 M urea, 2 M thiourea, and 2% sodium deoxycholate) was used. Protein samples were resolved by SDS/polyacrylamide gel electrophoresis under reducing condition and transferred to PVDF membranes. The blots were blocked with 10% skimmed milk, incubated with primary antibodies overnight at 4 °C, and incubated with secondary antibodies conjugated to horseradish peroxidase (1:10,000) (Jackson ImmunoResearch). The antibodies used were the following: anti-acetylated tubulin 1:1,000 (#T7451, Sigma-Aldrich); anti-ACTB 1:1,000 (#PM053, Medical & Biological Laboratories); anti-AKAP3 1:1,000 (#13907–1-AP, Proteintech); anti-BASIGIN 1:500 (#sc9757, Santa Cruz Biotechnology or #sc46700, Santa Cruz Biotechnology); anti-COX4 1:500 (#ab202554, Abcam); anti-FLAG 1:1,000 (#PM020, Medical & Biological Laboratories); anti-SPATA33 1:500; ant-GAPDH 1:500 (#sc25778, Santa Cruz Biotechnology); anti-PPP3CC 1:50 (#sc6122, Santa Cruz Biotechnology); anti-PPP3R2 1:50 (#sc6120, Santa Cruz Biotechnology); anti-PA 1:1,000 (#012–25863, FUJIFILM Wako Pure Chemical); anti-TOM20 1:500 (#sc11415, Santa Cruz Biotechnology); and anti-VDAC2 1:500 (#11663–1-AP, Proteintech). Anti-SPESP1 was previously generated in our laboratory (1:1,000) (44). Anti-1D4 was as described previously (45, 46) (1:5,000). The signals were detected using an ECL plus Western blotting detection kit (GE Healthcare). ImageJ (NIH) was used for densitometry.

### Immunofluorescence.

Spermatozoa were collected from the cauda epididymis, diluted in PBS, spotted onto slides, and air dried. The samples were then fixed with 4% paraformaldehyde for 10 min. After washing with PBS for 5 min, the slides were blocked with 5% bovine serum albumin and 10% goat serum in PBS for 1 h at room temperature. The slides were then incubated with anti-PA antibody (1:100, #012–25863, FUJIFILM Wako Pure Chemical) or anti-PPP3R2 antibody (1:100, #14005–1-AP, Proteintech) overnight at 4 °C and washed with PBS three times for 10 min each. After incubation with Alexa Fluor 546–conjugated secondary antibody (1:200, #A11081 or #A11071, Thermo Fisher Scientific) at room temperature for 2 h, the slides were washed with PBS three times for 10 min each. The slides were observed with an Olympus BX-53 microscope.

### Statistical Analysis.

A two-tailed Student’s *t* test was used for statistical analyses. Differences were considered significant at *P* < 0.05 (*) or highly significant at *P* < 0.01 (**) and *P* < 0.001 (***). Error bars are SD.

## Supplementary Material

Supplementary File

Supplementary File

Supplementary File

Supplementary File

Supplementary File

Supplementary File

Supplementary File

## Data Availability

All study data are included in the article and/or *SI Appendix*.
