# Supplementary material for: SPATA33 localizes calcineurin to the mitochondria and regulates sperm motility in mice
Source: Proc Natl Acad Sci U S A. 2021 Aug 26;118(35):e2106673118. doi: 10.1073/pnas.2106673118 (PMC8536318; doi:10.1073/pnas.2106673118)
Supplement: Supplementary File [file pnas.2106673118.sapp.pdf]

PNAS
www.pnas.org

Supplementary Information for

SPATA33 localizes calcineurin to the mitochondria and regulates sperm motility in mice

Haruhiko Miyata, Seiya Oura, Akane Morohoshi, Keisuke Shimada, Daisuke Mashiko, Yuki Oyama, Yuki Kaneda, Takafumi Matsumura, Ferheen Abbasi, Masahito Ikawa

Masahito Ikawa
Email: ikawa@biken.osaka-u.ac.jp

This PDF file includes:

Figures S1 to S9
Table S1
Legends for Movies S1 to S6

Other supplementary materials for this manuscript include the following:

Movies S1 to S6

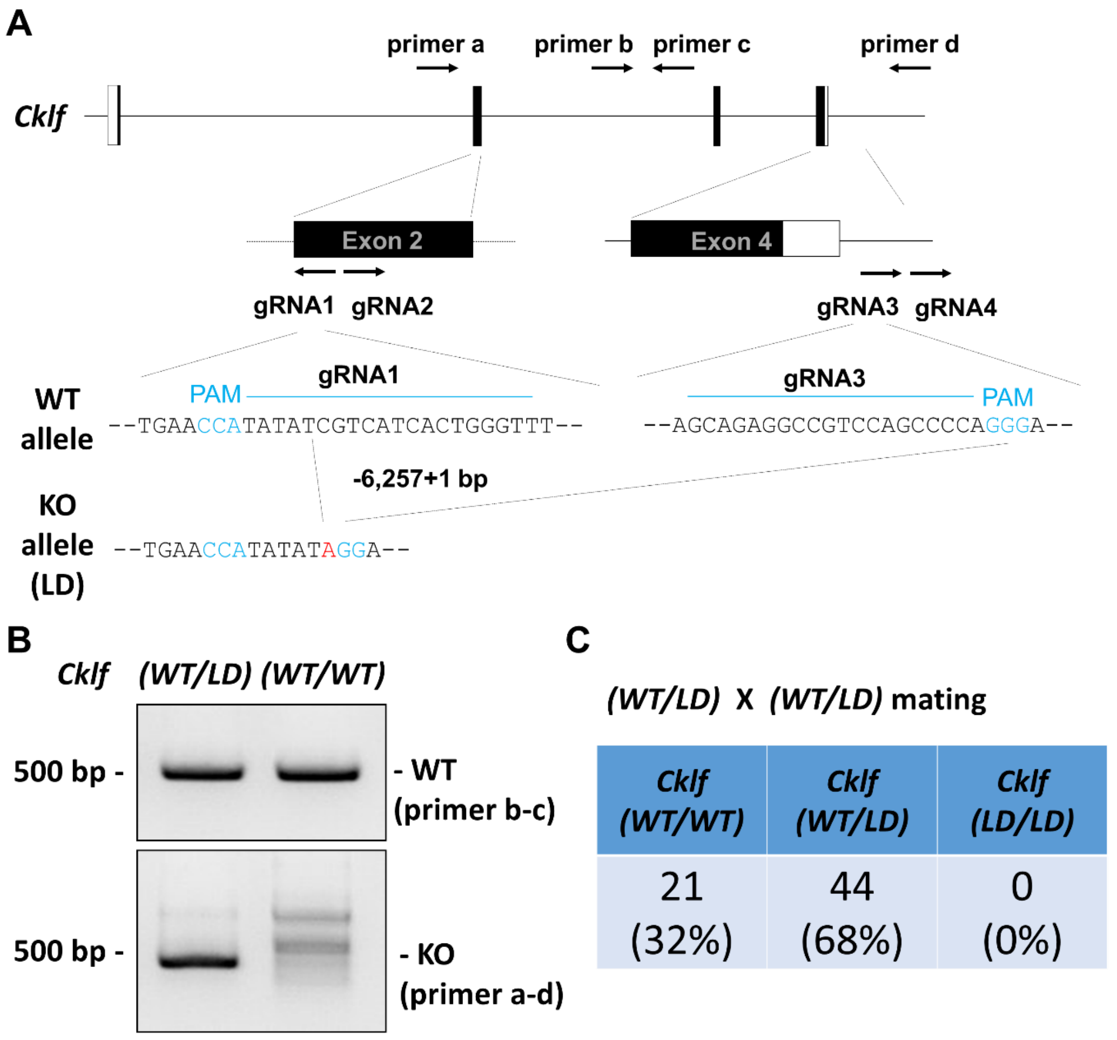

Fig. S1. Generation of *Cklf* large deletion (LD) mice.

(*A*) CRISPR/Cas9 targeting scheme. gRNAs were designed within exon 2 and intron 4. The sequence information of each gRNA is listed in SI Appendix, Table S1. PAM (protospacer adjacent motif) is shown in blue. Mice with 6,257 bp deletion plus 1 bp insertion (shown in red) were obtained. (*B*) Genotyping of *Cklf*$^{WT/LD}$ mice using primers shown in SI Appendix, Fig. S1A. (*C*) Offspring's genotypes from *Cklf*$^{WT/LD}$ x *Cklf*$^{WT/LD}$ mating. No *Cklf*$^{LD/LD}$ mice were obtained. The number of pups genotyped were shown. Offspring from three sets of mating (three females x one male) were analyzed.

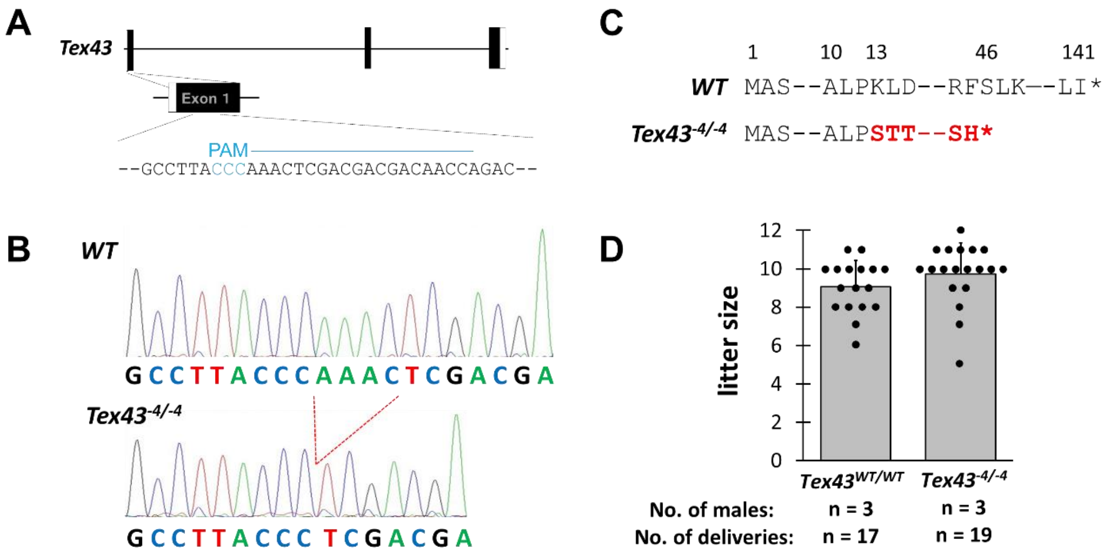

Fig. S2. Generation of *Tex43* indel mice.

(*A*) CRISPR/Cas9 targeting scheme. gRNA was designed within exon 1. PAM is shown in blue. (*B*) Wave pattern sequence of *Tex43* exhibits 4 bp deletion. (*C*) The 4 bp deletion caused a K13S mutation with a premature stop codon introduced 33 amino acids later. (*D*) Number of litters born per plug detected. *Tex43*$^{-4/-4}$ male mice were fertile.

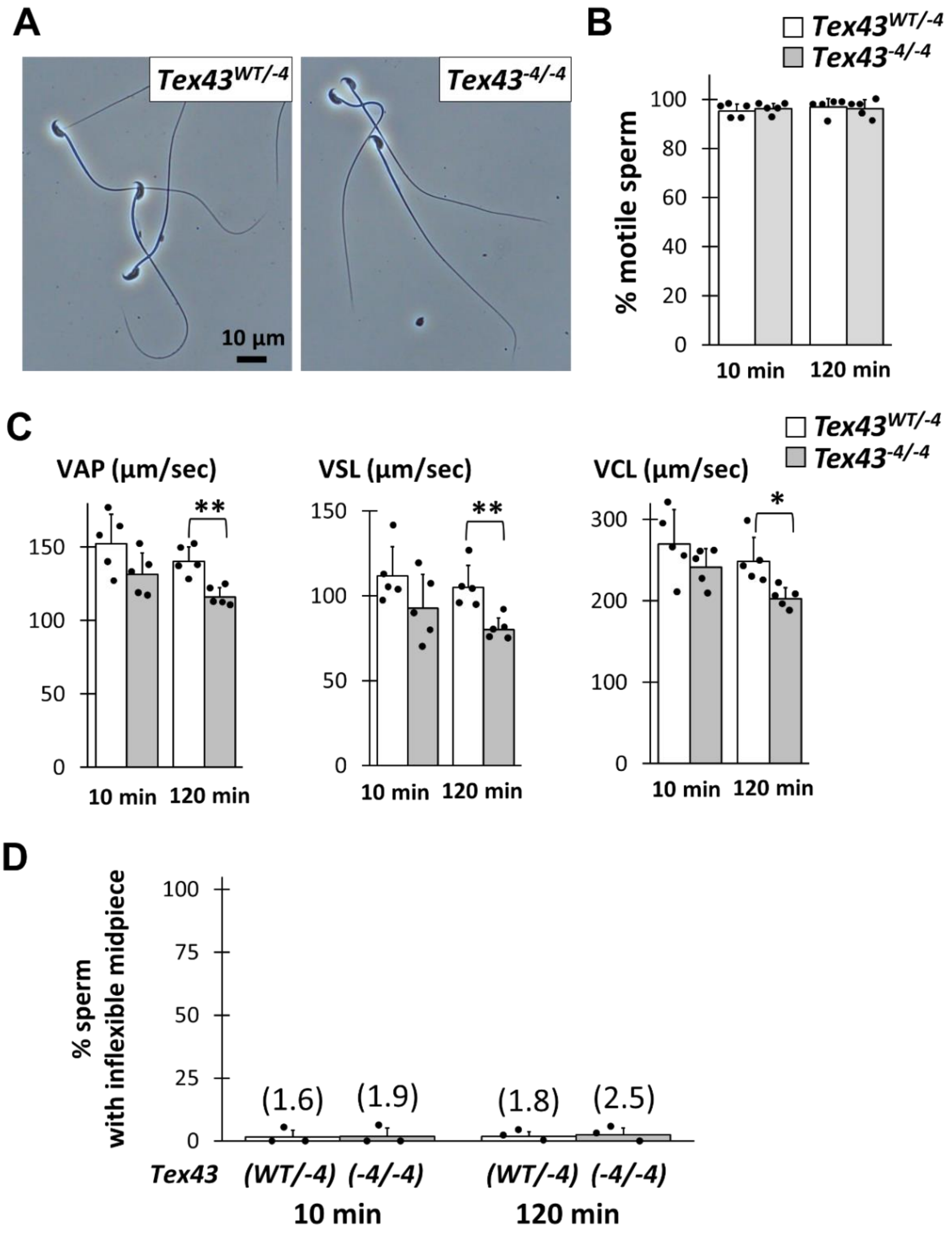

Fig. S3. Sperm morphology and motility of *Tex43*$^{-4/-4}$ male mice.

(*A*) Observation of spermatozoa obtained from cauda epididymis. No overt abnormalities were found in *Tex43*$^{-4/-4}$ mice. (*B*) Percentage of motile spermatozoa. n = 5 males each for *Tex43*$^{WT/-4}$ and *Tex43*$^{-4/-4}$ mice. (*C*) VAP (average path velocity), VSL (straight-line velocity), and VCL (curvilinear velocity) were analyzed. n = 5 males each for *Tex43*$^{WT/-4}$ and *Tex43*$^{-4/-4}$ mice. (*D*) The percentage of spermatozoa with an inflexible midpiece. n = 3 males each for *Tex43*$^{WT/-4}$ and *Tex43*$^{-4/-4}$ mice. Midpieces were flexible in *Tex43*$^{-4/-4}$ mice.

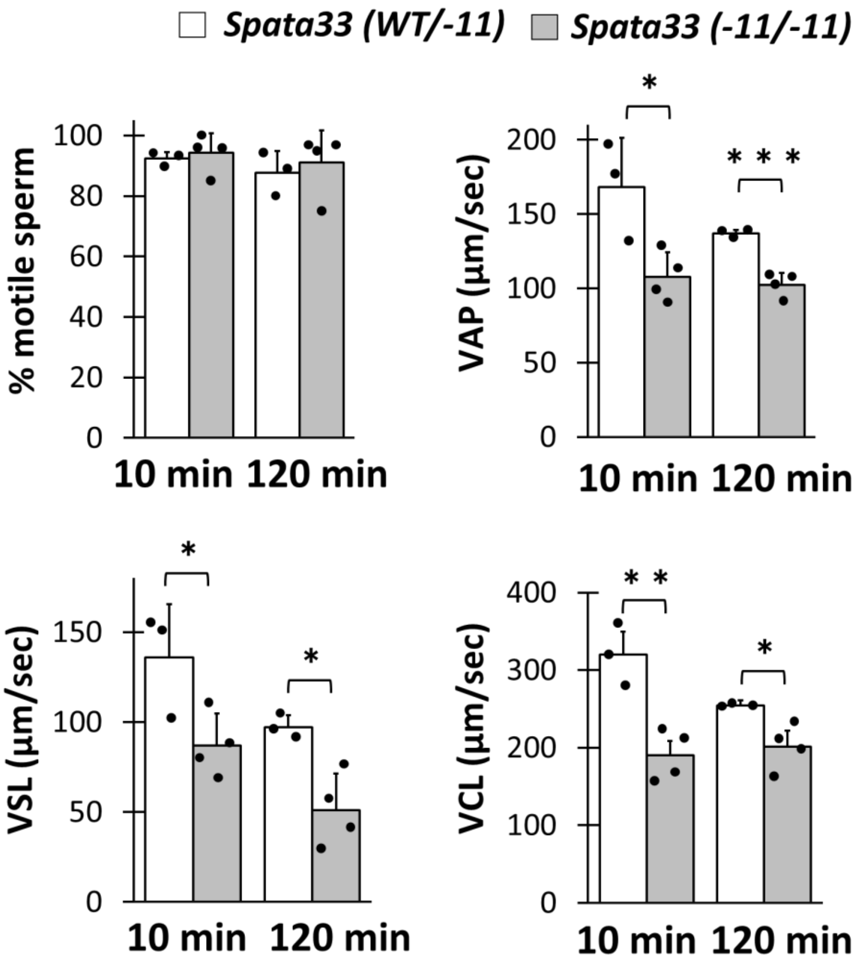

Fig. S4. Sperm motility of *Spata33*$^{-11/-11}$ mice.

Percentage of motile spermatozoa, VAP, VSL, and VCL were analyzed. n = 3 males for *Spata33*$^{WT/-11}$ and n = 4 males for *Spata33*$^{-11/-11}$ mice.

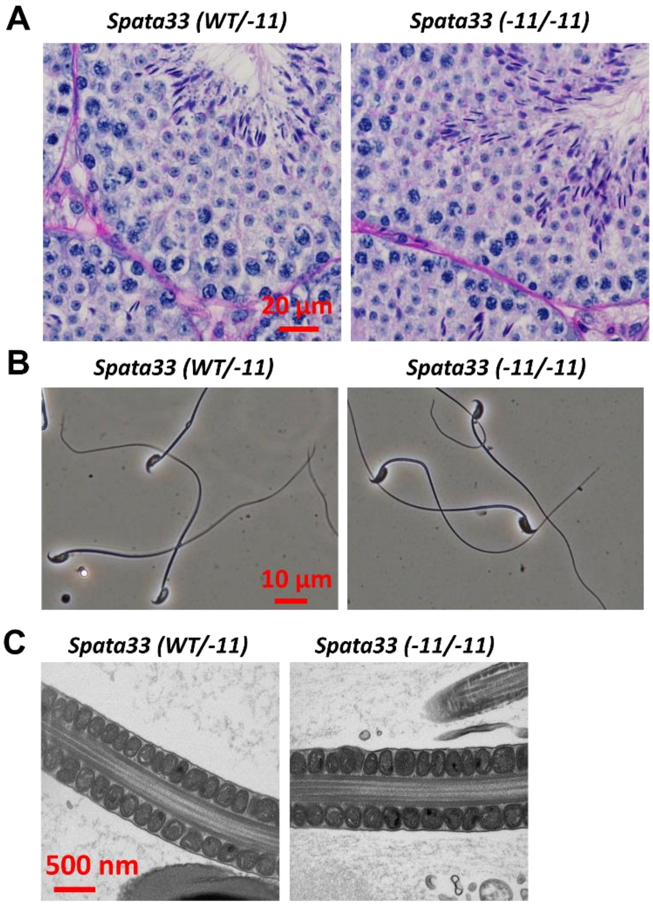

D

Litter size	*Spata33* (*WT/-11*)	*Spata33* (*-11/-11*)
0	0	99
1	0	1
2	0	1
3	0	2
4	0	1
5	0	1
6	0	2
7	2	5
8	1	5
9	9	4
10	5	2
11	1	1
12	5	0

Fig. S5. Testis morphology, sperm morphology, and litter size of *Spata33*$^{-11/-11}$ mice.
(*A*) PAS staining of testes. No overt abnormalities were found in *Spata33*$^{-11/-11}$ testis. (*B*) Observation of spermatozoa obtained from cauda epididymis. No overt abnormalities were found in *Spata33*$^{-11/-11}$ mice. (*C*) TEM analysis of spermatozoa. Longitudinal sections of the midpieces were shown. No structural abnormalities were observed in *Spata33*$^{-11/-11}$ mice. (*D*) Mating tests of *Spata33*$^{-11/-11}$ males. The numbers of each litter size obtained are shown. This result is summarized in Fig. 3B.

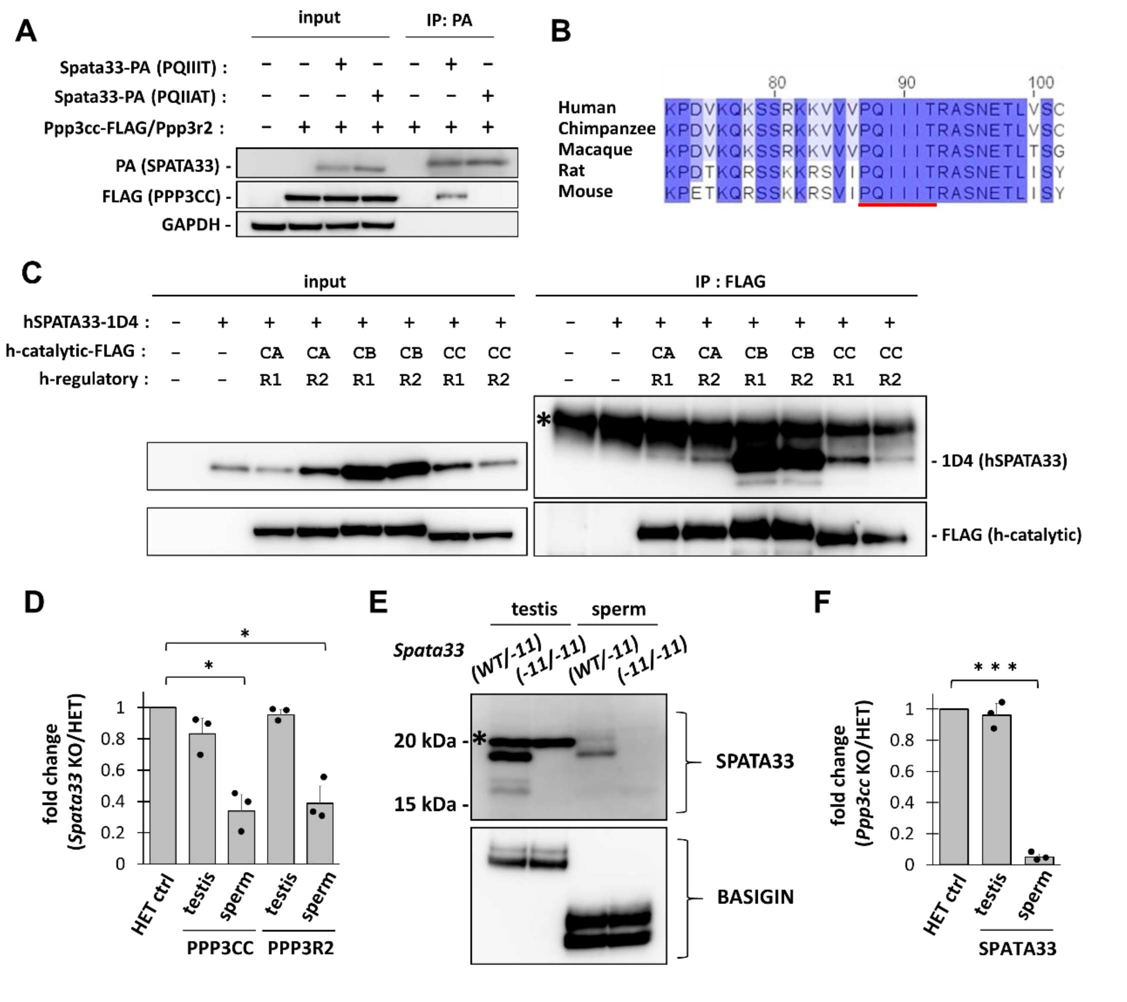

Fig. S6. Interaction of SPATA33 and sperm calcineurin.

(*A*) *Spata33-PA* (PQIIIT) or mutated *Spata33-PA* (PQIIAT) was co-expressed with *Ppp3cc-FLAG/Ppp3r2* in HEK293T cells and immunoprecipitation with PA antibody was performed. GAPDH was the control. (*B*) Parts of SPATA33 amino acid sequences in human, chimpanzee, macaque, rat, and mouse. The PQIIIT sequence is conserved, which is underlined in red. (*C*) Interaction of human SPATA33 and sperm calcineurin. Human 1D4-tagged SPATA33 was co-expressed with FLAG-tagged catalytic (PPP3CA, PPP3CB, or PPP3CC) and regulatory subunit (PPP3R1 or PPP3R2) of calcineurin. Cell lysates were immunoprecipitated with anti-FLAG antibody. The asterisk indicates the bands of IgG light chain. (*D*) Quantitative analysis of Fig. 4B. HET ctrl indicates the result of *Spata33*$^{WT/-11}$ mice that is used as standard. n = 3 males each for *Spata33*$^{WT/-11}$ and *Spata33*$^{-11/-11}$ mice. (*E*) Generation of SPATA33 antibody. The generated antibody detected one major band around 19 kDa as well as two minor bands in the testis around 17 kDa, and one major band around 19 kDa and one minor band around 20 kDa in the spermatozoa. These bands were depleted in *Spata33*$^{-11/-11}$ mice. BASIGIN was the control. The asterisk indicates a non-specific band. (*F*) Quantitative analysis of Fig. 4C. HET ctrl indicates the result of *Ppp3cc*$^{WT/-}$ mice that is used as standard. n = 3 males each for *Ppp3cc*$^{WT/-}$ and *Ppp3cc*$^{-/-}$ mice.

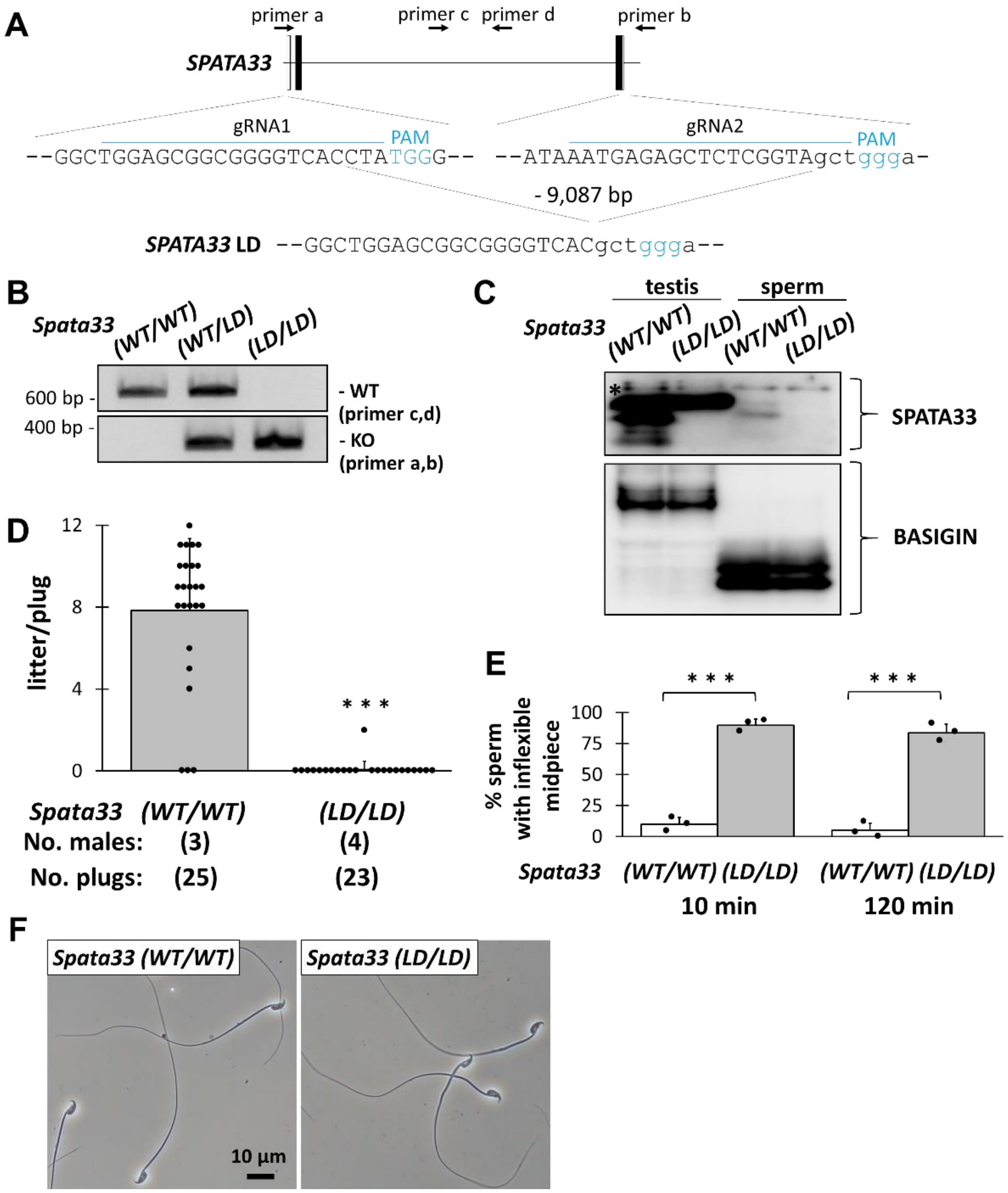

Fig. S7. Generation and analysis of *Spata33* LD mice.

(*A*) CRISPR/Cas9 targeting scheme. gRNAs were designed within exon 1 and exon 3. PAM is shown in blue. (*B*) Genotyping of *Spata33*$^{LD/LD}$ mice using primers shown in SI Appendix, Fig. S7A. (*C*) The same SPATA33 band pattern as *Spata33*$^{-11/-11}$ mice was detected in *Spata33*$^{LD/LD}$ mice. The band around 20 kDa in the testis (asterisk) was not depleted in *Spata33*$^{LD/LD}$ mice, indicating that this band is non-specific. (*D*) Number of litters born per plug detected. (*E*) The percentage of spermatozoa with an inflexible midpiece. n = 3 males each for *Spata33*$^{WT/WT}$ and *Spata33*$^{LD/LD}$ mice. Midpieces were inflexible in *Spata33*$^{LD/LD}$ mice. (*F*) Observation of spermatozoa obtained from the cauda epididymis. No overt abnormalities were found in *Spata33*$^{LD/LD}$ mice.

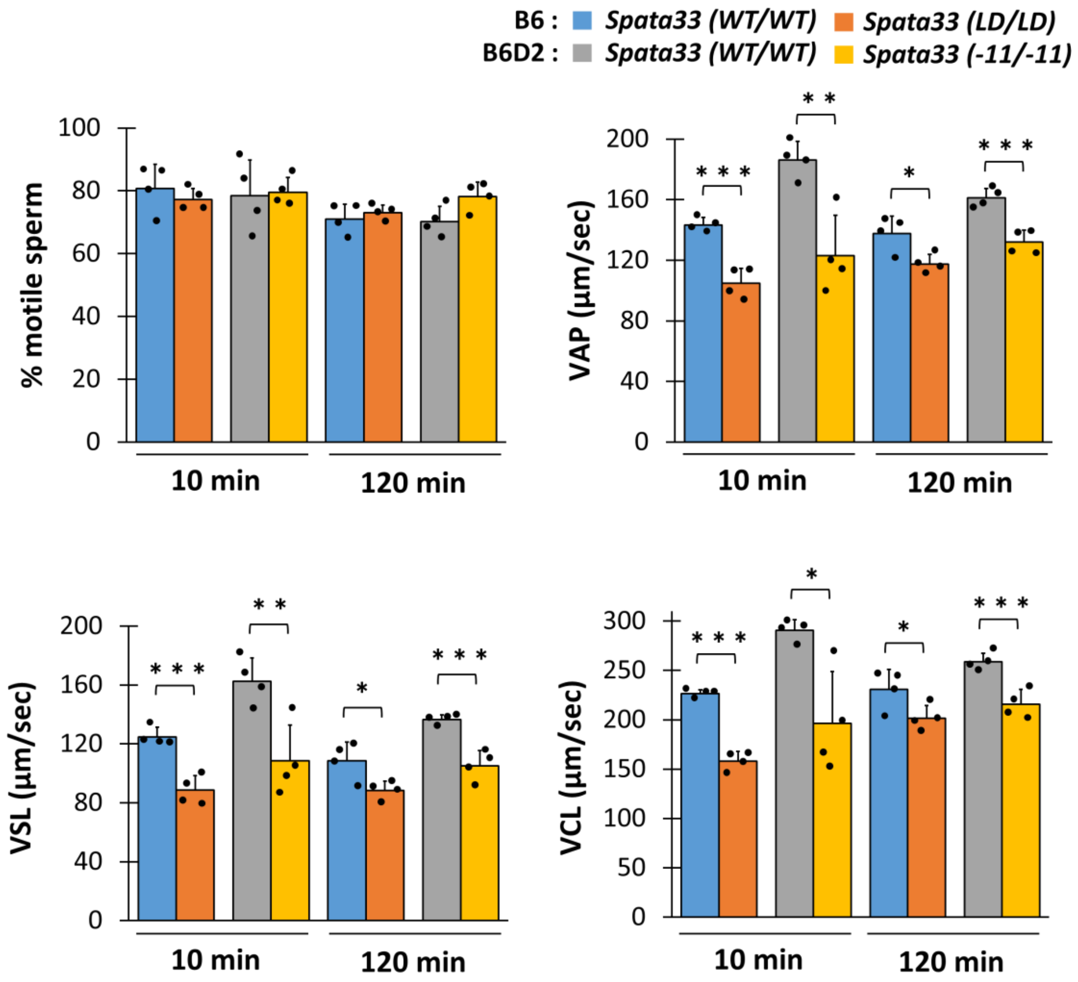

Fig. S8. Sperm motility of *Spata33*$^{-11/-11}$ and *Spatsa33*$^{LD/LD}$ mice.

Percentage of motile spermatozoa, VAP, VSL, and VCL were analyzed. n = 4 males each for *Spata33*$^{WT/WT}$ (C57BL/6N), *Spata33*$^{WT/WT}$ (B6D2), *Spata33*$^{LD/LD}$ (C57BL/6N), and *Spata33*$^{-11/-11}$ (B6D2) mice. There were significant differences in all the velocity parameters (VAP, VSL, VCL) when *Spata33*$^{WT/WT}$ (C57BL/6N) vs *Spata33*$^{LD/LD}$ (C57BL/6N) or *Spata33*$^{WT/WT}$ (B6D2) vs *Spata33*$^{-11/-11}$ (B6D2) were compared.

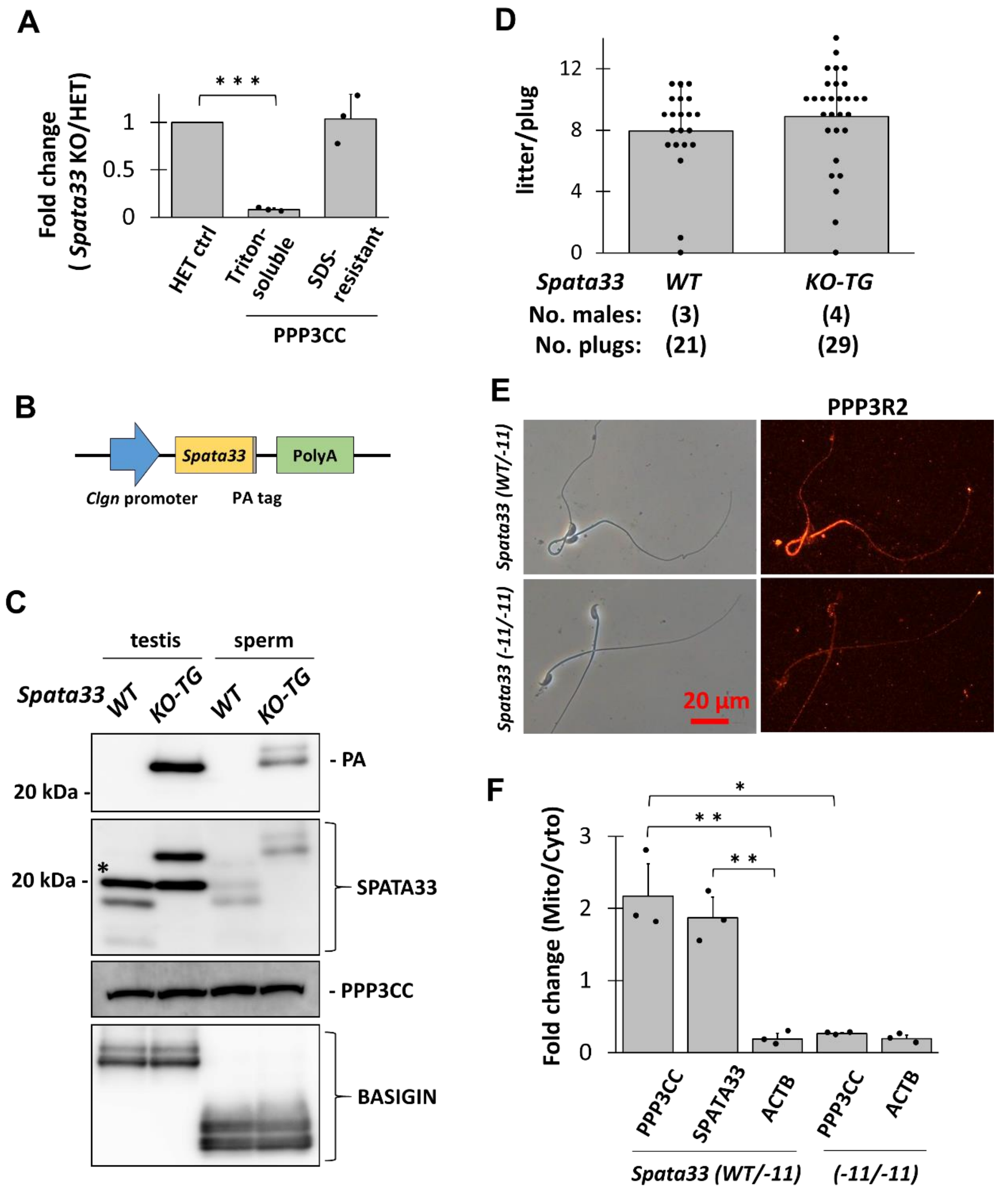

Fig. S9. Generation of *Spata33-PA* transgenic mice and analysis of PPP3CC, PPP3R2, and SPATA33 localization.

(*A*) Quantitative analysis of Fig. 5A. HET ctrl indicates the result of *Spata33*$^{WT/-11}$ mice that is used as standard. n = 3 males each for *Spata33*$^{WT/-11}$ and *Spata33*$^{-11/-11}$ mice. (*B*) The construct of transgene. *Spata33-PA* is expressed under the *Clgn* promoter. (*C*) SPATA33-PA can be detected using anti-PA or anti-SPATA33 antibody with Western blotting analysis. The transgene rescued the amount of PPP3CC in *Spata33*$^{-11/-11}$ mice. BASIGIN was the control. The asterisk indicates a non-specific band. (*D*) Number of litters born per plug detected. *Spata33-PA* transgene rescued the fertility of *Spata33*$^{-11/-11}$ male mice. (*E*) Localization of PPP3R2 in mature spermatozoa. Strong signal of PPP3R2 in the midpiece disappeared in *Spata33*$^{-11/-11}$ mice. (*F*) Quantitative analysis of Fig. 5D. n = 3 males each for *Spata33*$^{WT/-11}$ and *Spata33*$^{-11/-11}$ mice.

Table S1. Primer and gRNA sequences used in this study.

Primer or gRNA	Figure	Gene name	Sequence (5' to 3')
Primer	1c	*Cklf*	ATGGAGACTCCACGGCCGGTC
Primer	1c	*Cklf*	TTATTCTCTGTCATCGATGTCATTAGTAGACTTTTTCTG
Primer	1c	*Spata33*	GTTTTGTGAGTCGGTCGGG
Primer	1c	*Spata33*	CTTGAGTGCTTGGATGTCGC
Primer	1c	*Tex43*	CAGACAGCAGAGAACACATG
Primer	1c	*Tex43*	TAGTAGTGGGGTTCAAACCC
Primer	1c	*Actb*	AAGTGTGACGTTGACATCCG
Primer	1c	*Actb*	GATCCACATCTGCTGGAAGG
gRNA	Supple 1a	*Cklf*	AACCCAGTGATGACGATATA (gRNA1)
gRNA	Supple 1a	*Cklf*	TTCCTAGTCTTATACACGTG (gRNA2)
gRNA	Supple 1a	*Cklf*	GCAGAGGCCGTCCAGCCCCA (gRNA3)
gRNA	Supple 1a	*Cklf*	GGAGATCCCCAGCTGAGCGC (gRNA4)
Primer	Supple 1b	*Cklf WT*	TGAGCTACAGGATGAGTACC (primer b)
Primer	Supple 1b	*Cklf WT*	TGCTTGATGTGGATTGTCCG (primer c)
Primer	Supple 1b	*Cklf KO*	GACTCAGTACCCACGTTAGC (primer a)
Primer	Supple 1b	*Cklf KO*	GCCCGCTCCGGCAGCTATCC (primer d)
gRNA	Supple 2a	*Tex43*	TGGTTGTCGTCGTCGAGTTT
Primer	Supple 2b	*Tex43*	AAGAGCTCTGACCTGCATCC
Primer	Supple 2b	*Tex43*	TCCTCAGAGAAAAGTCTGCC
gRNA	2a	*Spata33*	GCGAACAACAGGCTTTCGGC
Primer	2b, 2c	*Spata33*	GGCTGTAGGTCTAACGGCTTGTCC
Primer	2b, 2c	*Spata33*	AGCCTAGCCTAGAGCCAAGG
Primer	4a	*Spata33*	TCTAGAGCCGCCATGGGCCAGTCGAAAAGC
Primer	4a	*Spata33*	GATATCTTCTGTGTTGTGTACATCATAGG
Primer	Supple 6c	Human *SPATA33*	TCTAGAGCTAGCGCCGCCATGGTGACGCACGCCGCTGG
Primer	Supple 6c	Human *SPATA33*	GAATTCTTCTTTGAGATGTGAATTATAGG
gRNA	Supple 7a	*Spata33*	TGGAGCGGCGGGGTCACCTA (gRNA1)
gRNA	Supple 7a	*Spata33*	AATGAGAGCTCTCGGTAGCT (gRNA2)
Primer	Supple 7b	*Spata33 WT*	AGCCTCTTGGCCATACACAG (primer c)
Primer	Supple 7b	*Spata33 WT*	CCCCATAGTGTGGTCGTAGC (primer d)
Primer	Supple 7b	*Spata33 KO*	TGGTAATGAGCTCCCGGAGC (primer a)
Primer	Supple 7b	*Spata33 KO*	CACCTGCTGTCTAGCACTGC (primer b)

Movie S1 (separate file). Sperm motility of *Tex43*$^{WT/-4}$ mice.

Sperm motility was videotaped at 200 frames per second 10 min after incubation. The movie is played at 20 frames/second (1/10 speed). The midpiece was flexible.

Movie S2 (separate file). Sperm motility of *Tex43*$^{-4/-4}$ mice.

Sperm motility was videotaped at 200 frames per second 10 min after incubation. The movie is played at 20 frames/second (1/10 speed). The midpiece was flexible.

Movie S3 (separate file). Sperm motility of *Spata33*$^{WT/-11}$ mice.

Sperm motility was videotaped at 200 frames per second 10 min after incubation. The movie is played at 20 frames/second (1/10 speed). The midpiece was flexible.

Movie S4 (separate file). Sperm motility of *Spata33*$^{-11/-11}$ mice.

Sperm motility was videotaped at 200 frames per second 10 min after incubation. The movie is played at 20 frames/second (1/10 speed). The midpiece was inflexible.

Movie S5 (separate file). Sperm motility of *Spata33*$^{WT/WT}$ mice.

Sperm motility was videotaped at 200 frames per second 10 min after incubation. The movie is played at 20 frames/second (1/10 speed). The midpiece was flexible.

Movie S6 (separate file). Sperm motility of *Spata33*$^{LD/LD}$ mice.

Sperm motility was videotaped at 200 frames per second 10 min after incubation. The movie is played at 20 frames/second (1/10 speed). The midpiece was inflexible.
